# Star-Shaped Glatiramer Acetate Mitigates Pulmonary Dysfunction and Brain Neuroinflammation in a Murine Model of Cryptococcus-Associated IRIS

**DOI:** 10.3390/biomedicines13112835

**Published:** 2025-11-20

**Authors:** Shehata Anwar, Jinyan Zhou, Lauren Kowalski, Joshua Saylor, Devanshi Shukla, Katelyn Boetel, Ziyuan Song, Kamal Sharma, Jianjun Cheng, Makoto Inoue

**Affiliations:** 1 Department of Comparative Biosciences, University of Illinois at Urbana-Champaign, 2001 South Lincoln Avenue, Urbana, IL 61802, USA; 2 Department of Pathology, Faculty of Veterinary Medicine, Beni-Suef University (BSU), Beni-Suef 62511, Egypt; 3 Neuroscience Program, University of Illinois at Urbana-Champaign, 405 North Matthews Avenue, Urbana, IL 61801, USA; 4 School of Molecular and Cell Biology, University of Illinois at Urbana-Champaign, 407 South Goodwin Avenue, Urbana, IL 61801, USA; 5 Department of Materials Science and Engineering, University of Illinois at Urbana-Champaign, 1304 West Green Street, Urbana, IL 61801, USA; 6 Department of Anatomy and Cell Biology, University of Illinois, Chicago, 808 S. Wood St., Chicago, IL 60612, USA; 7 School of Engineering, Westlake University, Hangzhou 310030, China; 8 Carl R. Woese Institute for Genomic Biology, 1206 West Gregory Drive, Urbana, IL 61801, USA; 9 Beckman Institute for Advanced Science and Technology, 405 North Matthews Avenue, Urbana, IL 61801, USA

**Keywords:** star-shaped glatiramer acetate, cryptococcus-associated IRIS (C-IRIS), neuroinflammation, pulmonary function, CD4^+^ T cells, microglia, immunotherapy

## Abstract

**Background: ***Cryptococcus*-associated immune reconstitution inflammatory syndrome (C-IRIS) is a life-threatening complication of immune recovery, often triggered by antiretroviral therapy and characterized by Th1-skewed CD4^+^ T cell hyperactivation, neuroinflammation, and pulmonary dysfunction. **Methods:** Using a validated murine model of unmasking C-IRIS, we assessed the therapeutic potential of star-shaped glatiramer acetate (sGA), a structurally enhanced derivative of the FDA-approved immunomodulator glatiramer acetate (GA). sGA was administered intraperitoneally on days 1 and 3 post-CD4^+^ T cell reconstitution. **Results:** sGA significantly ameliorated C-IRIS-associated respiratory dysfunction, including increasing breaths per minute by ~35% and improved minute volume, total respiratory cycle time, expiration time, and inspiration time. Survival rate grew to 75% on day 14 for sGA-treated C-IRIS mice. In both the lung and the brain, sGA reduced total CD4^+^ T cells and selectively diminished Th1 cells by 50–60% and Th17 cells by 40–50%. Activated microglia decreased by 45% within the brain, indicating attenuated innate immune activation. Golgi-Cox analysis revealed region-specific neuroprotection: neuronal loss in the prefrontal cortex, lateral hypothalamus, and periaqueductal gray was rescued by 25–40%, whereas hippocampal neurons were relatively preserved, and basolateral amygdala neurons showed no significant recovery. **Conclusion:** Collectively, our findings suggest that sGA exerts neuroprotection in C-IRIS by limiting peripheral CD4^+^ T cell effector activity and suppressing CNS-resident immune activation. This study supports the use of sGA as a promising preclinical therapeutic candidate for C-IRIS and other Th1-mediated neuroinflammatory conditions.

## 1. Introduction

*Cryptococcus*-associated IRIS (C-IRIS) is a severe clinical complication marked by an exaggerated inflammatory response following rapid immune restoration, most commonly observed in immunocompromised individuals, such as HIV patients initiating antiretroviral therapy (ART) or patients with underlying opportunistic infections. This hyperinflammatory state, driven predominantly by an influx of CD4^+^ T cells skewed toward Th1 and Th17 subtypes, results in significant morbidity, manifesting as neurological symptoms (e.g., headaches, memory impairment, and lesion-induced edema) and non-neurological complications, including pulmonary dysfunction [1,2,3]. Epidemiological data indicate that 10–45% of HIV patients develop C-IRIS, with up to 60% of cases occurring within the first month of ART initiation, underscoring the urgent need for effective therapeutic interventions. Conditions that subject a person to a dramatic shift in immune composition, such as pregnancy and autoimmune diseases, are also recognized risk factors for IRIS [4]. Symptomatically, headaches, fever, memory loss, and lesion-induced edema are typical in patients [1,3,5]. Additionally, they may exhibit non-neurological symptoms, such as pulmonary disease [6,7,8,9,10,11].

C-IRIS is associated with a robust CD4^+^ T cell response, skewed toward the Th1 subtype in affected patients. Our previously established murine model of C-IRIS, which mimics the unmasking form of the syndrome, has been instrumental in elucidating the immunopathogenesis of this condition [12,13]. In this model, immunocompromised Rag1^−/−^ mice, lacking T and B cells, are intranasally infected with *Cryptococcus neoformans* (Cn) serotype A H99 (CnH99), followed by intravenous CD4^+^ T cell reconstitution three weeks post-infection. This results in a robust Th1-mediated inflammatory response, characterized by systemic upregulation of proinflammatory cytokines, cerebral immune infiltration, leading to neurodegeneration and pulmonary dysfunction [13]. These findings highlight the critical role of dysregulated CD4^+^ T cell responses in driving C-IRIS pathology.

Current management strategies for C-IRIS, including nonsteroidal anti-inflammatory drugs and corticosteroids, are limited by their potential to increase susceptibility to secondary infections and cause drug–drug interactions [14]. Corticosteroid use in C-IRIS has also been linked to delayed fungal clearance and poor long-term outcomes in some populations [2]. Glatiramer acetate (GA), also known as Copolymer 1 or Copaxone, is a synthetic amino acid copolymer and disease-modifying therapy (DMT) used to treat multiple sclerosis (MS) [15]. It is an injectable immunomodulator medication approved by the FDA to reduce the frequency of relapses in relapsing-remitting MS [15,16]. GA has also been effectively used in the treatment of clinically isolated syndrome (CIS) [17]. Moreover, early subcutaneous injection of GA in APP/PS1 mice, a model of Alzheimer’s disease (AD), delayed disease progression, reduced amyloid beta plaque burden, regulated neuroinflammation, promoted neuroprotection, and induced dendritic-like microglia [18,19]. GA suppresses Th1 cell differentiation, interferes with the antigen-presenting function of specific immune cells, and consequently increases the secretion of anti-inflammatory cytokines [20,21]. Additionally, GA-specific T helper cells migrate to the brain and suppress neuroinflammation [22]. Star-shaped GA (sGA) is a modified polymer form of GA with a spherical, three-dimensional structure. sGA function is similar to GA, but has more rapid cellular uptake, improved tissue retention, and increased potency in vivo, as demonstrated in a mouse model of MS [23].

Building on these properties, we investigated the therapeutic effect of sGA in our established mouse model of C-IRIS. We found that sGA significantly suppresses C-IRIS development via reductions in CD4^+^ T cell and Th1 cell populations in both lungs and brain and neuronal damage in the brain.

## 2. Materials and Methods

### 2.1. Mice

C57BL/6J and *Rag1^−/−^* (strain #002216) mice were purchased from The Jackson Laboratory for use in this study. All animals were housed in specific pathogen-free (SPF) conditions in groups, maintained on a 12-h light/dark cycle, and provided with standard pelleted rodent chow and water ad libitum. *Rag1^−/−^* mice used for C-IRIS induction were 16–20 weeks old, while CD4^+^ T-cell donor mice were 6- to 8-week-old C57BL/6J mice. All experimental mice were littermates derived from the same dams, age-matched across groups, and randomly assigned to treatment conditions to minimize variability. Male and female mice were randomly assigned for experimentation, as no significant sex-based differences were noted in our previous investigations. All animal procedures were conducted in accordance with protocols approved by the Institutional Animal Care and Use Committee (IACUC) at the University of Illinois (protocol number 22140).

### 2.2. C-IRIS Induction

C-IRIS was induced following established protocols [12,13]. CnH99 (ATCC 208821) was cultured on yeast extract-peptone-dextrose (YPD) agar plates or in YPD liquid medium (1% yeast extract, 2% peptone, 2% dextrose) at 30 °C with constant shaking at 200 rpm overnight. Mice were anesthetized with isoflurane (5% for induction and 3% for maintenance) in 2 L/min oxygen and intranasally inoculated with 100 CnH99 yeast cells suspended in 30 μL phosphate-buffered saline (PBS). Three weeks post-infection, CD4^+^ T cell reconstitution was performed via intravenous injection of 1 × 10^6^ CD4^+^ T cells suspended in 200 μL PBS supplemented with 2% fetal bovine serum (FBS). CD4^+^ T cells were isolated from the spleens and inguinal/axillary lymph nodes of naïve C57BL/6J mice (6–8 weeks of age) using a two-step magnetic selection protocol. Initially, non-CD4^+^ populations, including B cells, CD8^+^ T cells, dendritic cells, neutrophils, and macrophages, were eliminated via negative selection using biotin-conjugated antibodies against CD19 (Cat# 115504, BioLegend, San Diego, CA, USA), CD11c (Cat# 117304, BioLegend, San Diego, CA, USA), CD8 (Cat# 100704, BioLegend, San Diego, CA, USA), Ly6G (Cat# 127604, BioLegend, San Diego, CA, USA), and CD11b (Cat# 101204, BioLegend, San Diego, CA, USA), followed by magnetic separation with the EasySep™ Mouse Streptavidin RapidSpheres™ Isolation Kit (Cat# 19860A, STEMCELL Technologies, Cambridge, MA, USA). Subsequently, CD4^+^ T cells were positively selected using a biotin-conjugated anti-CD4 antibody (Cat# 100404, BioLegend, San Diego, CA, USA) in combination with the EasySep™ Biotin Positive Selection Kit (Cat# 17665, STEMCELL Technologies, Cambridge, MA, USA). Following reconstitution, mice were monitored daily.

### 2.3. Drug Administration

Based on established kinetics in the C-IRIS model, where pathological CD4^+^ T cell infiltration begins on day 1 and peaks between days 3 and 5 [13], sGA was administered intraperitoneally at a dose of 1 mg/kg (0.1 mL per 10 g body weight) in sterile PBS, as previously described. Treatment was initiated following CD4^+^ T cell transfer for C-IRIS induction, with two dosing time points on days 1 and 3 post-reconstitution to coincide with early T cell activation and maximize therapeutic effect. Mice were randomly assigned to receive either vehicle (PBS) or sGA treatment.

### 2.4. Pulmonary Function Assessment

Respiratory function was evaluated using a whole-body plethysmography (WBP) system (Buxco Small Animal Whole-Body Plethysmography; Data Sciences International). Experimental groups (*n* = 9–13 mice/group) included sGA-treated C-IRIS mice, vehicle-treated C-IRIS mice, and naïve controls. Mice were acclimated to the testing environment before data acquisition. Recordings were performed without anesthesia or physical restraint, with each session lasting 5 min. Between trials, the plethysmography chambers were thoroughly cleaned with 70% ethanol to eliminate residual odors and prevent cross-contamination. Respiratory parameters were analyzed using Ponemah^®^ software, version 5.2 (Data Sciences International). For the survival assay, mice (*n* = 16–18 per group) were monitored daily for 14 days following CD4^+^ T-cell reconstitution, and humane endpoints were applied in accordance with our approved IACUC protocol.

### 2.5. Brain Harvesting and Golgi-Cox Neuronal Staining

Mice (*n* = 5 mice/group) were deeply anesthetized with isoflurane and perfused transcardially with ice-cold 1× Tris-buffered saline (TBS), followed by 4% paraformaldehyde (PFA). Brains were rapidly extracted and processed using the FD Rapid Golgi-Stain Kit (Cat. PK401, FD NeuroTechnologies, Columbia, MD, USA) according to the manufacturer’s protocol. Briefly, each brain was immersed in 10 mL of a 1:1 mixture of Solutions A and B (containing mercuric chloride, potassium dichromate, and potassium chromate) for 24 h and then incubated in a freshly prepared A + B solution for an additional 14 days in the dark. The tissue was subsequently transferred to Solution C for 24 h, then replaced with fresh Solution C, and incubated for an additional 3 days. After impregnation, brains were embedded in Tissue-Tek^®^ OCT compound, and 60 μm-thick coronal sections were cut and stored in 0.02% sodium azide at 4 °C. Sections were mounted on poly-L-lysine–coated slides and imaged under bright-field illumination with a Hamamatsu NanoZoomer digital slide scanner equipped with a 20× objective. Neuronal density was quantified in predefined brain regions.

### 2.6. Flow Cytometric Analysis of Brain and Lung Immune Cells

Flow cytometric analysis was conducted seven days following C-IRIS induction to assess immune cell populations in the brain and lungs of mice (5 mice/group). Tissues were harvested and digested in collagenase D (1 mg/mL in PBS) at 37 °C, then filtered through a 70 μm cell strainer and centrifuged at 277× *g* for 10 min at 4 °C. Mononuclear cells were isolated using a 30%/70% Percoll density gradient by centrifugation at 377× *g* for 20 min at room temperature. Fc receptors were blocked using Fc Block (BD Pharmingen) for 7 min on ice, followed by surface staining with fluorochrome-conjugated antibodies (1:200) for 30 min on ice. For intracellular cytokine analysis, cells were fixed and permeabilized using the BD Cytofix/Cytoperm Fixation/Permeabilization Kit (Cat# 554714, BD Biosciences, Franklin Lakes, NJ, USA) before staining. Data acquisition was performed using a Cytek Aurora flow cytometer, and the data were analyzed with FCS Express software Version 7 (De Novo Software, Pasadena, CA, USA). Antibodies used included APC-Fire 750 anti-CD45 (Cat# 103154, BioLegendSan, Diego, USA), PE/Cy5 anti-CD3 (Ref# 15-0031-82, eBioscience, San Diego, CA, USA), PE/Cy7 anti-CD4 (Cat# 100422, BioLegend, San Diego, CA, USA), FITC anti-IFNγ (Cat# 505806, BioLegend, San Diego, CA, USA), PE anti-IL-17 (Cat# 506904, BioLegend, San Diego, CA, USA), and PE anti-CD11b (Cat# 101208, BioLegend, San Diego, CA, USA). A standardized sequential gating strategy was applied. First, forward and side scatter (FSC/SSC) were used to exclude debris. Doublets were excluded by plotting FSC-H versus FSC-A. Viable single cells were gated by excluding cells positive for a viability dye. CD45^+^ leukocytes were selected, and CD3^+^CD4^+^ T cells were identified from this gate. Th1 and Th17 subsets were then detected by intracellular cytokine staining for IFN-γ and IL-17, respectively, following stimulation with PMA/ionomycin and brefeldin A. Microglia were identified based on their expression profile of CD11b^+^CD45^low^, distinguishing them from infiltrating peripheral myeloid cells CD11b^+^CD45^high^. For accurate compensation, single-stained beads were used for each fluorophore, and fluorescence minus one controls were included to define positive gates. Flow cytometry was performed using a Cytek Aurora spectral cytometer (Cytek Biosciences, Bethesda, Fremont, CA, USA), and data were analyzed using FCS Express software Version 7 (De Novo Software, Pasadena, CA, USA).

### 2.7. Statistical Analyses

All statistical analyses were conducted using GraphPad Prism version 9 (GraphPad Software, La Jolla, CA, USA). Group comparisons were performed using two-tailed unpaired Student’s *t*-tests, one-way ANOVA followed by Tukey’s post hoc test, or the log-rank (Mantel–Cox) test for survival analysis, as appropriate. A *p*-value ≤ 0.05 was considered statistically significant. All experiments were conducted blindly and randomly. No animals or data points were excluded from analysis. The number of biological replicates (*n*) and exact *p*-values are provided in the figure legends.

## 3. Results

### 3.1. sGA Treatment Improves Respiratory Dysfunction

**Figure 1:**
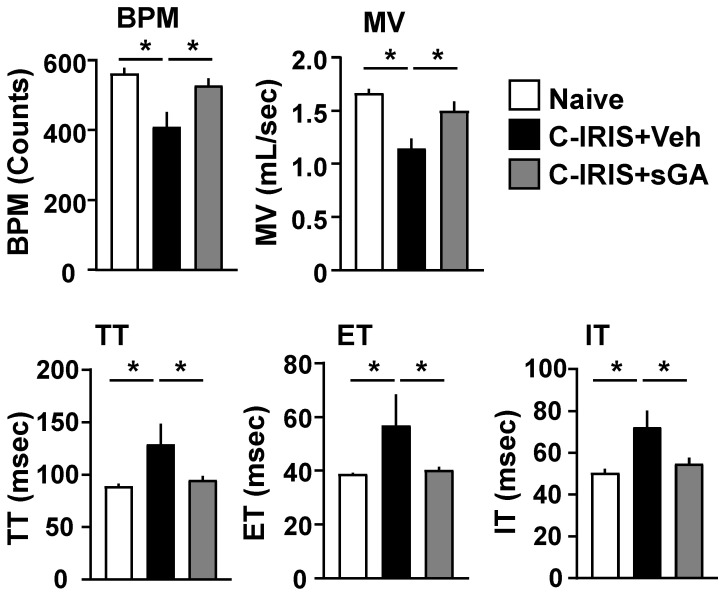
sGA preserves pulmonary function in C-IRIS mice. Whole-body plethysmography was performed five days after CD4^+^ T cell reconstitution to assess respiratory function in naïve *Rag1^−/−^* (naïve) mice, vehicle (PBS)-treated *Cryptococcus*-associated IRIS (C-IRIS) mice, and star-shaped GA (sGA)-treated C-IRIS mice. Measured parameters included breaths per minute (BPM), total respiratory cycle time (TT), expiration time (ET), inspiration time (IT), and minute volume (MV). *n* = 9–13 mice per group. Data are presented as mean ± SEM. Asterisks denote statistical significance (* *p* < 0.05) by one-way ANOVA followed by Tukey’s post hoc test.

To assess the therapeutic potential of sGA in the context of C-IRIS, we first examined its effect on pulmonary function. Respiratory parameters were measured using WBP five days post-CD4^+^ T cell transfer. To induce C-IRIS disease, mice were intranasally inoculated with 100 yeast cells of CnH99 and i.v. transferred CD4^+^ T cells (10^6^ cells) isolated from naïve mice at three weeks after CnH99 infection [12,13]. sGA treatment paradigm (1 mg/kg, administered twice) was shown to be effective in a mouse model of MS, a disease mediated principally by CD4^+^ T cells [23]. Since our model of C-IRIS is similarly driven by pathogenic CD4^+^ T cells, we applied the identical dosing regimen of sGA in our C-IRIS studies to assess its therapeutic effect. Compared to naïve controls, C-IRIS mice exhibited hallmark features of pulmonary dysfunction, including a significant reduction in breaths per minute (BPM) and minute volume (MV), alongside prolonged total respiratory cycle time (TT), expiration time (ET), and inspiration time (IT). Strikingly, intraperitoneal administration of sGA significantly mitigated these abnormalities. All measured respiratory parameters in sGA-treated C-IRIS mice showed significant improvement relative to those in vehicle-treated C-IRIS mice, with several returning to levels comparable to those of naïve controls (Figure 1). These data indicate that sGA effectively restores pulmonary function.

### 3.2. sGA Reduces Mortality in the C-IRIS Model

**Figure 2:**
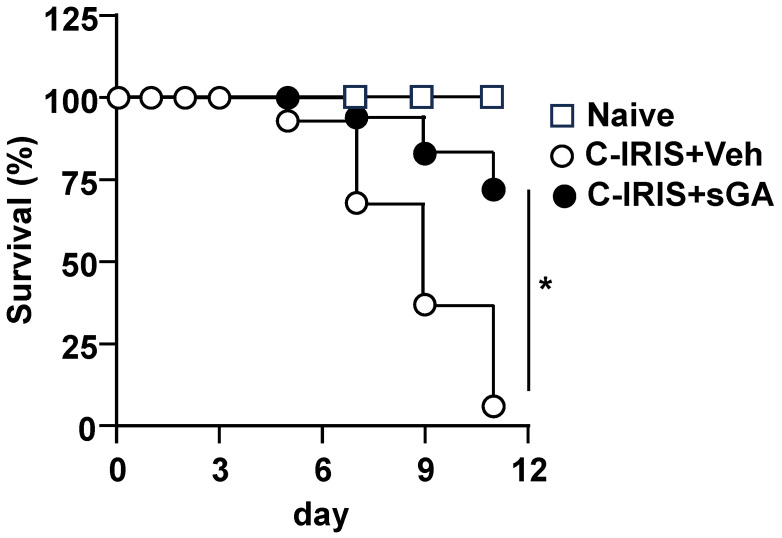
sGA treatment improves survival in C-IRIS mice. Kaplan–Meier survival curves were generated over 14 days following CD4^+^ T cell reconstitution in naïve *Rag1^−/−^* (naïve) controls, vehicle (PBS)-treated *Cryptococcus*-associated IRIS (C-IRIS) mice, and star-shaped GA (sGA)-treated C-IRIS mice. *n* = 16–18 mice per group. Data were analyzed using the log-rank (Mantel–Cox) test. * *p* < 0.05.

Given the known association between respiratory failure and mortality in C-IRIS, we next conducted a Kaplan–Meier survival analysis. Vehicle-treated C-IRIS mice exhibited a sharp decline in survival, beginning on day six post-reconstitution, with mortality reaching 100% by day 14. In contrast, sGA treatment significantly prolonged survival, demonstrating a clear protective effect against disease lethality (Figure 2).

### 3.3. sGA Suppresses Brain and Pulmonary CD4^+^ T Cell Responses and Microglial Expansion

**Figure 3:**
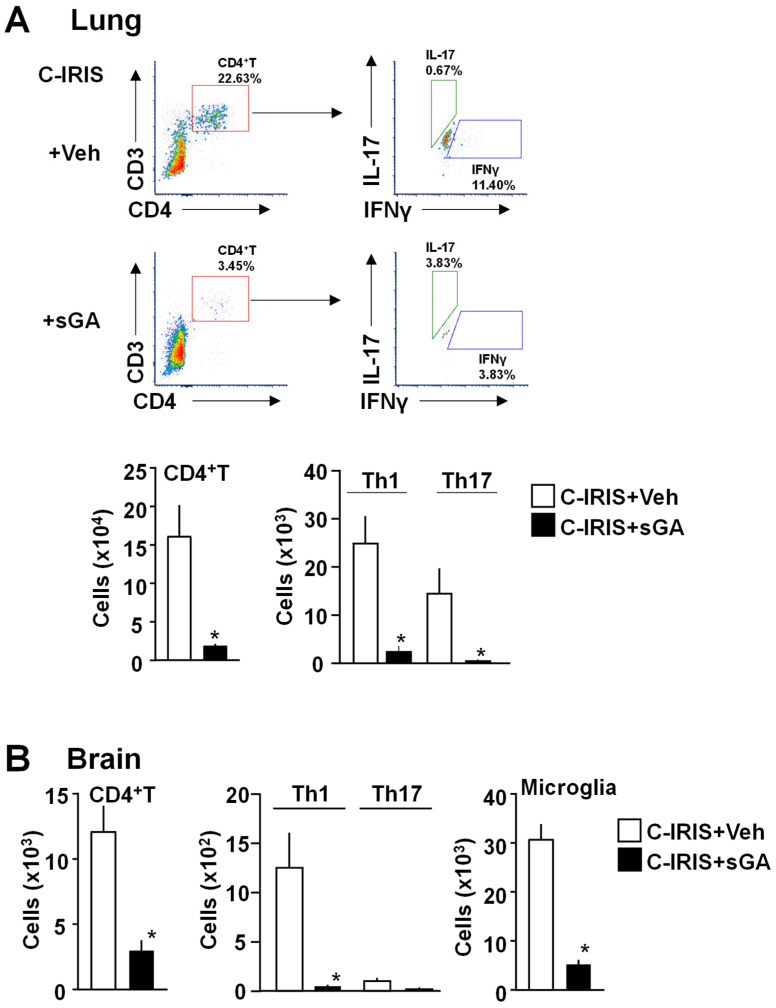
sGA reduces inflammatory cell populations in the lungs and brains of C-IRIS mice. (**A**) Flow cytometry analysis of CD4^+^ T cells and their Th1 (IFNγ^+^) and Th17 (IL-17^+^) subsets in lung tissue from vehicle (PBS)-treated *Cryptococcus*-associated IRIS (C-IRIS) and star-shaped GA (sGA)-treated C-IRIS mice. (**B**) Flow cytometry analysis of CD4^+^ T cells, Th1, Th17 subsets, and CD11b^+^ microglia in brain tissue across the same groups. *n* = 5 mice per group. Data is presented as mean ± SEM. Statistical analysis was performed using one-way ANOVA followed by Tukey’s post hoc test. * *p* < 0.05.

Because C-IRIS disease is mediated by Th1 cells in the brain [12,13], sGA suppresses Th1 differentiation [20,21]. We therefore conducted flow cytometry at 7 days after C-IRIS induction to evaluate the Th1 cell population in the brain and lungs. In the lung, vehicle-treated C-IRIS mice exhibited CD4^+^ T cells, including Th1 and Th17 subsets (Figure 3A). Treatment with sGA resulted in a marked reduction in total CD4^+^ T cells, as well as in Th1 and Th17 populations (Figure 3A). In the brain, vehicle-treated C-IRIS mice also demonstrated an enrichment of Th1 and Th17 cells within the CD4^+^ T cell population, which was significantly diminished following sGA treatment (Figure 3B). Additionally, microglia count was significantly lower in sGA-treated mice compared to vehicle-treated C-IRIS mice (Figure 3B).

### 3.4. sGA Protects Against C-IRIS Neurodegeneration

**Figure 4:**
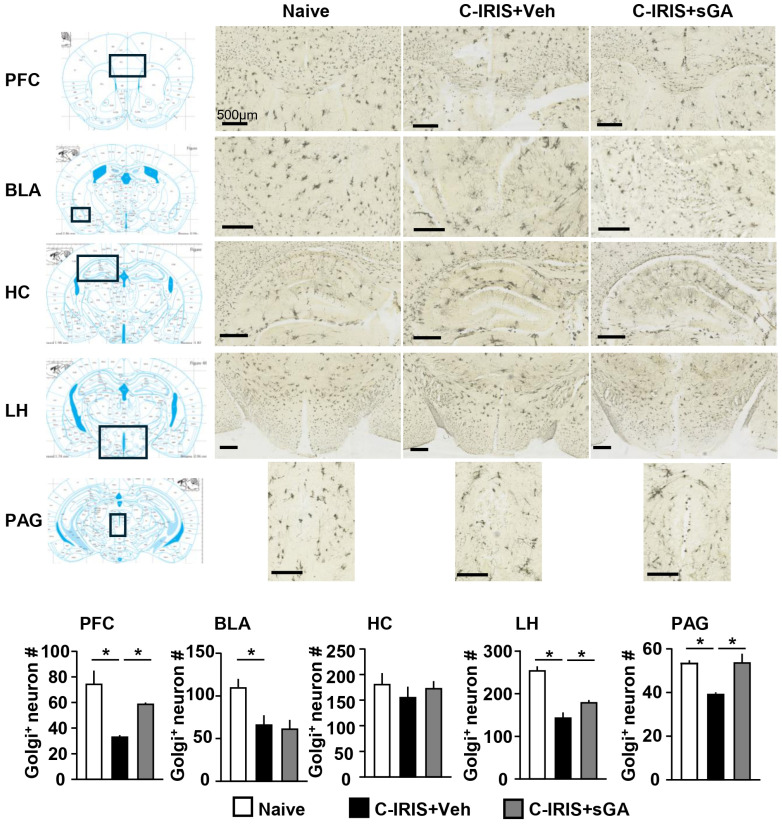
sGA protects against C-IRIS neurodegeneration. Golgi-Cox staining was used to visualize and quantify neurons in the prefrontal cortex (PFC, AP = 1.54 mm, ML = −1 to 1 mm, and DV = 1 to 2), basolateral amygdala (BLA, AP = −0.94 mm, ML = 2.5 to 3.2 mm and −2.5 to −3.2 mm, and DV = 4.3 to 5.0), hippocampus (HC, AP = −1.82 mm, ML = 0 to 2.5 mm and −2.5 to 0 mm, and DV = 1 to 2.4), lateral hypothalamus (LH, AP = −2.06 mm, ML = −1.5 to 1.5 mm, and DV = 4.3 to 6), and periaqueductal gray (PAG, AP = −2.70 mm, ML = −0.5 to 0.5 mm, and DV = 2.8 to 4) from naïve *Rag1^−/−^* (naïve) mice, vehicle (PBS)-treated *Cryptococcus*-associated IRIS (C-IRIS) mice, and star-shaped GA (sGA)-treated C-IRIS mice. The Mouse Brain in Stereotaxic Coordinates by Keith B.J. Franklin and George Paxinos [24] was used as reference for identifying the selected brain regions, indicated by the black boxes in the figure. Golgi-Cox-stained neuron numbers (Golgi^+^ neurons #) were evaluated. *n* = 5 mice per group. Images were acquired with a Hamamatsu NanoZoomer digital slide scanner using a 20× objective. Scale bar = 500 μm. Data are presented as mean ± SEM. Statistical comparisons were performed using one-way ANOVA with Tukey’s post hoc test. * *p* < 0.05.

The murine C-IRIS condition is characterized by neurodegeneration, resulting from the infiltration of CD4^+^ T cells into the brain tissue, which possess neurotoxic properties [13], leading to neurogenic pulmonary dysfunction. In addition, C-IRIS patients have multiple neurological symptoms, including headache, memory loss, meningeal irritation signs, and visual disturbance [5], likely mediated by neurodegeneration in the brain. Thus, we quantified neuron numbers in various brain regions that may be involved in the neurological symptoms of C-IRIS. Additionally, we assessed neuronal status in the brains of C-IRIS mice following sGA treatment. As shown in Figure 4, Golgi-Cox-stained neuron numbers were significantly reduced in the prefrontal cortex (PFC), basolateral amygdala (BLA), lateral hypothalamus (LH), and periaqueductal gray (PAG) of C-IRIS mice, while no significance was observed in the hippocampus. Notably, sGA treatment significantly mitigated neuronal loss in the PFC, LH, and PAG (Figure 4). This neuroprotective effect supports the therapeutic potential of sGA in mitigating C-IRIS-associated neurodegeneration.

## 4. Discussion

C-IRIS is a life-threatening condition occurring upon rapid recovery of immune competence, particularly in HIV-infected individuals initiating ART. It is characterized by excessive CD4^+^ T cell activation, particularly Th1-driven responses, leading to neuroinflammation, pulmonary dysfunction, and mortality [2,13]. Both clinical and experimental studies have identified excessive Th1-skewed CD4^+^ T cell responses as key drivers of C-IRIS, promoting IFNγ–mediated microglial activation and neuronal damage [12,13,25]. In line with clinical reports of pulmonary involvement in C-IRIS patients [6,9], our murine model exhibited substantial respiratory dysfunction, as evidenced by altered plethysmographic parameters, including BPM and MV, along with prolonged respiratory cycle times (ET, IT, TT). These features recapitulate pulmonary pathophysiology previously described in experimental C-IRIS [13].

Clinical and experimental evidence support the central role of CD4^+^ T cells, especially Th1-polarized subsets, in the development of C-IRIS. In HIV-positive patients starting ART, immune reconstitution often triggers an overwhelming Th1-driven inflammatory response against residual fungal antigens, leading to severe clinical deterioration despite previous infection control [2,25]. This pathological process has been replicated in mouse models of C-IRIS, where the adoptive transfer of CD4^+^ T cells into CnH99-infected Rag1^−/−^ mice causes rapid disease onset, characterized by increased Th1 cell expansion, immune cell infiltration into the brain, and innate immune activation in both the brain and lungs [12,13]. Notably, the proliferation and activation of CD4^+^ T cells in this setting may be Cn antigen-specific, demonstrated by a significant increase in CD4^+^ T cells, up to tenfold in the lungs of infected mice after reconstitution [12]. This expansion coincides with the upregulation of co-stimulatory molecules, such as CD80/CD86, on dendritic cells, indicating active antigen presentation and suggesting that T cell activation relies largely on Cn antigens [13]. These results highlight the significance of antigenic context in driving C-IRIS immunopathology. GA, including its star-shaped form, sGA, is thought to exert its therapeutic effects through multiple mechanisms. One primary mode is competitive binding to MHC class II molecules on antigen-presenting cells, which prevents the presentation of pathogenic antigens and subsequent T-cell priming [21,26,27]. This mechanism is particularly relevant in C-IRIS, where T cell overactivation occurs due to persistent exposure to fungal antigens. By occupying MHC class II, GA may decrease the magnitude and duration of CD4^+^ T cell responses, reducing the cytokine storm that causes tissue damage. Additionally, GA encourages the differentiation of naïve CD4^+^ T cells into Th2 and regulatory subsets, rather than proinflammatory Th1 cells [20,28]. This shift toward an anti-inflammatory T cell profile could further explain the observed reduction in neuroinflammation and clinical improvements seen after sGA treatment in our model. Past studies in MS and Alzheimer’s models have shown that GA induces protective T cell subsets capable of migrating to the CNS and promoting local neuroprotection [22,29]. Microglia are activated by cytokines such as IFN-γ and GM-CSF from infiltrating T cells, contributing to inflammation through antigen presentation and oxidative stress [30]. Similar effects of GA on microglial suppression have been reported in EAE and AD models [19,29]. Our findings suggest that similar mechanisms may be at work in C-IRIS, and that sGA might restore immune balance through both peripheral effects and direct actions within the CNS. Thus, both sGA and GA emerge as promising candidates for targeted immune-modulation in C-IRIS, especially for patients where non-selective immunosuppression carries additional risks.

The broad range of CNS symptoms observed in C-IRIS patients highlights the brain’s vulnerability to immune-mediated damage and its potential responsiveness to immunomodulatory treatments. Cognitive impairment, confusion, personality changes, and memory deficits are frequently seen in clinical cases of C-IRIS and strongly suggest involvement of the frontal lobe and limbic system [2,5]. In our mouse model, we observed a significant reduction in neurons in the PFC, a region crucial for working memory, attention regulation, and emotional control [31]. These results imply that neurodegeneration specifically in the PFC may directly contribute to the neuropsychiatric symptoms seen in C-IRIS, which often complicate clinical management and lead to poor patient outcomes [1]. Additionally, we observed significant neuronal loss in the PAG, a brainstem area involved in descending pain modulation and autonomic functions [32]. Disruption of this circuitry may explain the increased reports of chronic pain and dysautonomia in C-IRIS patients. Although pain in HIV and cryptococcosis has traditionally been linked to peripheral mechanisms or medication side effects, increasing evidence indicates a central component mediated by inflammation affecting midbrain pain control centers [5]. Importantly, treatment with sGA significantly increased neuronal counts in both the PFC and PAG, suggesting this compound may provide region-specific neuroprotection. This supports earlier findings that GA and its derivatives can modulate neuroinflammation, promote microglial shifts toward neuroprotective states, and prevent neuronal loss in models of MS and AD [19,23,29]. The capacity of sGA to inhibit Th1/Th17-driven pathology and microglial activation may foster a CNS environment that favors neuronal survival and synaptic health. While our current focus is on neuronal density, sGA may also help preserve neuronal structure and connectivity, including dendritic arborization, spine morphology, and synaptic density, features that are impacted in neuroinflammatory conditions even without outright cell loss [19,28]. Future research involving ultrastructural analysis and behavioral tests, such as novel object recognition or pain threshold assays, will be crucial to confirm these functional benefits. Collectively, these findings support that sGA could serve as a dual-action therapy in C-IRIS by modulating peripheral immune responses while simultaneously providing CNS protection. This holds important clinical significance, as current IRIS treatments (e.g., corticosteroids) often suppress inflammation broadly and pose risks of opportunistic infections and relapse [14]. A targeted immunomodulatory agent, such as sGA, might offer a safer and more effective alternative.

Region-specific neurodegeneration in the brains of C-IRIS mice provided crucial insights into the cellular susceptibility in C-IRIS and the therapeutic effects of sGA. Using Golgi-Cox staining, we saw a significant decrease in neuron numbers in the PFC, LH, and PAG of C-IRIS mice, which was notably improved by sGA treatment. Conversely, no significant neuron loss was detected in the hippocampus, and no protective effect of sGA was observed in the BLA. This likely reflects region-specific CD4^+^ T cell infiltration, as earlier studies have shown that Th1 CD4^+^ T cells are the main mediators of neuron damage in C-IRIS [12,13]. Still, the preservation of neuron health in the hippocampus despite systemic immune activation might be due to limited CD4^+^ T cell recruitment or local immune privilege. On the other hand, the BLA could be vulnerable to different disease pathways not driven by Th1 cells. Notably, GM-CSF^+^ CD4^+^ T cells, which have been linked to neuroinflammation in other models, such as EAE and viral encephalitis, may play a role in BLA’s vulnerability [33,34]. Our previous work demonstrated that disease severity was only partially reduced using *Ifng^−/−^* CD4^+^ T cells, suggesting a role for non-Th1 effector pathways [13]. Notably, while total neuron counts remained stable in some regions, this does not rule out the possibility of subtle structural damage. Changes in dendritic structures, synaptic spine density, and neurite complexity are early signs of neuroinflammatory injury and may not be detected by Golgi-Cox staining-based measurements alone [19,29]. Therefore, future research utilizing high-resolution imaging and morphometric analysis will be crucial to determine whether sGA offers protection at the subcellular level in brain areas without apparent cell loss. Also, future studies will incorporate behavioral assessments to determine whether the observed neuroprotection translates into functional improvements.

C-IRIS manifests clinically in two distinct forms: unmasking and paradoxical. The unmasking form occurs when a previously subclinical cryptococcal infection becomes overt after immune reconstitution, whereas paradoxical IRIS refers to clinical deterioration despite prior antifungal treatment and initial symptom resolution [2]. The current study uses a murine model that reflects the unmasking phenotype, characterized by CD4^+^ T cell reconstitution without prior antifungal therapy. Despite different clinical contexts, both forms of C-IRIS could potentially have similar immunopathogenic mechanisms, namely, a rapid and dysregulated Th1-dominant immune response upon ART initiation [33,34]. Given GA’s potent immunomodulatory capacity, particularly its ability to suppress pathogenic Th1 responses, it may also hold therapeutic potential in paradoxical C-IRIS. Future research evaluating sGA in paradoxical C-IRIS models or clinical settings could be beneficial to explore the broader potential of sGA across various IRIS phenotypes.

This study also has limitations that define important directions for future research. Because a validated paradoxical C-IRIS model that incorporates antifungal therapy has not yet been established in the field, our work relied on the standard unmasking model, which captures only one of the two major clinical phenotypes. The development of an antifungal-treated paradoxical model is underway in our laboratory and will allow broader evaluation of therapeutic candidates and a direct comparison between the clinical phenotypes. The lack of FDA-approved therapy and established standard-of-care medication for C-IRIS means no pharmacological control can be reliably used. In addition, we did not perform direct comparisons with linear GA or corticosteroids, as these agents require distinct dosing strategies and, in the case of corticosteroids, induce broad immunosuppression that would interfere with the CD4^+^ T cell-dependent mechanisms central to C-IRIS. Pharmacokinetic, biodistribution, and CNS bioavailability analyses were not undertaken, and dose–response studies remain to be defined. Our previous study on sGA pharmacokinetics demonstrated high hydrolytic stability in plasma and enhanced efficacy with low dosage. However, such work in the context of C-IRIS will be essential for optimizing sGA dosing and for establishing quantitative links between tissue exposure and therapeutic efficacy. Our immunological assessments were focused on pathogenic Th1 and Th17 CD4^+^ T cells and microglia, leaving the roles of other immune populations, such as T regulatory cells, GM-CSF-producing T cells, and systemic cytokine responses, to be addressed in future studies. Moreover, although we demonstrated regional neuronal protection, we did not evaluate ultrastructural neuronal changes or perform behavioral testing, and determining whether the observed neuroprotection translates into functional improvement remains an important next step. Finally, while all sGA-treated mice remained clinically healthy following two low-dose injections, formal acute or chronic toxicology studies were not conducted, and given that rare hepatotoxicity has been reported with repeated administration of clinical GA [35], a comprehensive safety evaluation of sGA will be required. These considerations collectively outline a clear framework for advancing mechanistic understanding, optimizing dosing, establishing safety, and ultimately supporting translational development of sGA for C-IRIS.

## 5. Conclusions

This study demonstrates that sGA ameliorates multiple pathological features of C-IRIS, including respiratory dysfunction, mortality, tissue-infiltrating inflammatory T cells, microglial expansion, and regional neuronal loss. These effects were achieved through early systemic administration of sGA. The findings suggest that sGA holds promise as a novel immunomodulatory therapy for IRIS and warrants further investigation in mechanistic and translational studies.

## Data Availability

The data supporting the findings of this study are available from the corresponding author upon request. Due to privacy and confidentiality considerations, the data are stored on our lab server and are not publicly available, but they will be shared with qualified researchers upon request. The Mouse Brain in Stereotaxic Coordinates by Keith B.J. Franklin and George Paxinos was used as reference for identifying brain regions.
